# Conserved role of the urotensin II receptor 4 signalling pathway to control body straightness in a tetrapod

**DOI:** 10.1098/rsob.210065

**Published:** 2021-08-11

**Authors:** Faredin Alejevski, Michelle Leemans, Anne-Laure Gaillard, David Leistenschneider, Céline de Flori, Marion Bougerol, Sébastien Le Mével, Anthony Herrel, Jean-Baptiste Fini, Guillaume Pézeron, Hervé Tostivint

**Affiliations:** ^1^ Physiologie moléculaire et adaptation UMR 7221 CNRS and Muséum National d'Histoire Naturelle, Paris, France; ^2^ Mécanismes adaptatifs et évolution UMR 7179 CNRS and Muséum National d'Histoire Naturelle, Paris, France

**Keywords:** urotensin II, *Xenopus*, spinal cord, cerebrospinal fluid-contacting neurons, muscles, scoliosis

## Abstract

Urp1 and Urp2 are two neuropeptides of the urotensin II family identified in teleost fish and mainly expressed in cerebrospinal fluid (CSF)-contacting neurons. It has been recently proposed that Urp1 and Urp2 are required for correct axis formation and maintenance. Their action is thought to be mediated by the receptor Uts2r3, which is specifically expressed in dorsal somites. In support of this view, it has been demonstrated that the loss of *uts2r3* results in severe scoliosis in adult zebrafish. In the present study, we report for the first time the occurrence of *urp2,* but not of *urp1*, in two tetrapod species of the *Xenopus* genus. In *X. laevis*, we show that *urp2* mRNA-containing cells are CSF-contacting neurons. Furthermore, we identified *utr4,* the *X. laevis* counterparts of zebrafish *uts2r3,* and we demonstrate that, as in zebrafish, it is expressed in the dorsal somatic musculature. Finally, we reveal that, in *X. laevis,* the disruption of *utr4* results in an abnormal curvature of the antero-posterior axis of the tadpoles. Taken together, our results suggest that the role of the Utr4 signalling pathway in the control of body straightness is an ancestral feature of bony vertebrates and not just a peculiarity of ray-finned fishes.

## Graphical abstract

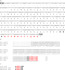

## 1. Introduction

The urotensin II (UII) family is a multigenic family of neuropeptides, evolutionarily related to somatostatin [1], which consists of four paralogous genes called *uts2*, *uts2-related peptide* (*urp*, also called *uts2b* in mammals and *uts2d* in fishes), *urp1* and *urp2* [2,3]. While *uts2* and *urp* exist both in fish and in tetrapods, so far, *urp1* and *urp2* have only been found in ray-finned fishes (actinopterygians) [2,3]. All peptides of this family act through a canonical family of G protein-coupled receptors called urotensin II receptors (Utr or Uts2r) [3]. The occurrence of multiple *utr* genes (*utr1*–*utr5*) has been reported in non-mammalian vertebrate genomes [3–5], which is in contrast with the single gene (*utr1*) present in mammals [3,6–9].

Since its discovery in 1980 [10], UII has prompted a large number of studies and it has been reported to regulate many physiological processes in the CNS and peripheral tissues, such as sleep, anxiety, depression, food intake, locomotion, neuroendocrine action, osmoregulation, cardiovascular functions and immunity (see [11] for review). In contrast with those of UII, the functions of Urps are much less understood. However, recent studies in zebrafish (*Danio rerio*) have shown that Urp1 and Urp2 play a critical role in spine morphogenesis [12–14].

*urp1* and *urp2* genes have been first characterized from the Japanese eel (*Anguilla japonica*) [15] and zebrafish [16], respectively, and were subsequently detected in all teleost species investigated, as well as in the spotted gar (*Lepisosteus oculatus*), a ray-finned fish species that diverged from teleosts before the teleost-specific whole-genome duplication [2,3]. In zebrafish, *urp1* and *urp2* are primarily expressed in the spinal cord and the hindbrain. In the spinal cord, their transcripts mainly co-localize in a small population of sensory neurons called cerebrospinal fluid (CSF)-contacting neurons [16,17].

The view that Urp1 and Urp2 are required for correct axis formation and maintenance in zebrafish is supported by several lines of evidence: (i) *urp2* and *urp1* expression is strongly affected in various zebrafish mutants sharing a curved, instead of a straight, body axis feature [12–14]; (ii) in zebrafish embryos, knock-down of *urp1* leads to a curled down axis, while its overexpression leads to the opposite curvature [12]; (iii) zebrafish mutant for *uts2r3 (*initially called *uts2ra),* a member of the family of the urotensin II receptors specifically expressed in dorsal muscles, results in severe spine deformations in adult [12].

The morphogenetic defects caused by deregulation of the Urp1/2-Uts2r3 pathway in zebrafish are clearly reminiscent of some of the manifestations of idiopathic scoliosis (IS) in humans [12,13], a complex genetic disorder characterized by three-dimensional spinal curvatures [18,19]. Recently, zebrafish has emerged as a powerful system for studying IS, owing to well-developed genetic resources and a natural susceptibility to spinal curvature [20]. The studies cited above obviously belong within this context. However, the main question that arises is to what extent their results can be extrapolated to humans.

Today, it is well recognized that neither *urp1* nor *urp2* are present in mammals. By contrast, whether these peptides exist in other tetrapods has never been investigated to date. In the present study, we report for the first time the characterization of the *urp2* (but not *urp1*) gene in two closely related tetrapod species, the western clawed frog (*Xenopus tropicalis*) and the African clawed frog (*X. laevis*). We demonstrate that *X. laevis urp2* mRNA, as in zebrafish, mainly occurs in CSF-contacting neurons of the spinal cord and hindbrain. We also show that *X. laevis utr4*, the counterpart of zebrafish *uts2r3*, is primarily expressed in dorsal somites (see table 1 for the UII receptor nomenclature used n this article). Finally, we reveal that the gene knock-out of *utr4* results in an abnormal curvature of the antero-posterior axis of the tadpoles that impacts their locomotion. Taken together, our results strongly suggest that the role of the Utr4 signalling pathway in the control of body straightness is an ancestral feature of bony vertebrates (osteichthyes) and not just a peculiarity of ray-finned fishes.

**Table 1.** Nomenclature of urotensin II receptors.

	nomenclature according to Tostivint *et al*. (2014)	UTR1	UTR2	UTR3	UTR4	UTR5
*X. tropicalis*	Ensembl nomenclature	urotensin 2 receptor (uts2r)	absent	urotensin-2 receptor-like 2 (uts2rl2)	urotensin-2 receptor-like 3 (uts2rl3)	urotensin 2 receptor-like (uts2rl)
	NCBI ID	BBC20764		BBC20765	BBC20766	BBC20767
	Ensembl ID	ENSXETG00000012229		ENSXETG00000015991	ENSXETG00000019036	ENSXETG00000030401
zebrafish	Ensembl nomenclature	urotensin 2 receptor (CABZ01027646.1)	urotensin 2 receptor 5 (uts2r5)	urotensin 2 receptor 4 (uts2r4)	urotensin 2 receptor 3 (uts2r3)	urotensin 2 receptor- like (LO017791.1)
	NCBI ID	XP_009305031.1	XP_001334493.1	XP_005172122.3	XP_005157985.1	XP_021336025.1
	Ensembl ID	ENSDARG00000009624	ENSDARG00000096870	ENSDARG00000096322	ENSDARG00000040816	ENSDARG00000115189

## 2. Material and methods

### 2.1. Animals

Animal husbandry *X. laevis* (outbred, wild type) were obtained from the National Biological Resource Center in Rennes (France). Animals were maintained at 23°C until stage NF52-55 then were sacrificed. Embryos were obtained by *in vitro* fertilization using wild-type *X. laevis* according to [21].

### 2.2. Molecular cloning of *Xenopus* Urp2 cDNA

A genomic sequence from *X. tropicalis* potentially encoding a *urp*2-like sequence (ACFWKYCIQNK) was recently reported [22]. Based on this nucleotide sequence, first primers and nested primers were designed to amplify the 5′-end of an *X. laevis urp*2 cDNA using the Advantage 2 PCR kit (Clontech). 5′RACE-ready cDNAs were constructed from 1 µg of poly(A^+^) RNA using the SMARTer RACE cDNA Amplification kit (Clontech), as previously described [23]. PCR was carried out in a MyCycler thermal cycler (Bio-Rad) under the following conditions: initial denaturation at 95°C/2 min, 5 cycles of 94°C/30 s and 72°C/2 min; 5 cycles of 94°C/30 s, 70°C/1 min and 72°C/1 min; 25 cycles of 94°C/30 s 68°C/1 min and 72°C/1 min; and the final extension at 72°C/7 min. The primers used were Xla_*urp*2 Rev x Universal Primer A Mix (UPM) then Xla_*urp*2 Rev Nest x Nested Universal Primer A Mix (NUP) (see electronic supplementary material, table S1 for primer sequences). PCR products were subcloned into the pGEM-T vector (Promega, Charbonnières-les-Bains, France) and Sanger sequenced (eurofinsgenomics.eu). A Blast search of the *X. laevis* genome database (available in Xenbase, http://www.xenbase.org) was performed using the 5′RACE *X. laevis urp2* sequence as a query. This search allowed us to detect two *X. laevis urp2* genes, called *urp*2S and *urp*2 L. Based on the genomic DNA sequences, four pairs of primers (Xla_*urp*2S For, Xla_*urp*2S Rev, Xla_*urp*2 L For and Xla_*urp*2 L Rev; see electronic supplementary material, table S1 for primer sequences) were designed to amplify the corresponding cDNA sequences. The basic cycling conditions of the PCRs were set as follows: initial denaturation at 94°C/2 min, 35 cycles of 94°C/30 s, gene-specific annealing for 45 s and 72°C/60 s and the final extension at 72°C for 7 min. PCR products were sequenced as described above. The coding sequence of the two *X. laevis urp*2 cDNAs has been deposited in the GenBank database under the accession numbers MZ054702 and MZ054703 for *urp2L* and *urp2S*, respectively. Data from *X. laevis* were used to identify the full-length *X. tropicalis urp2* cDNA and to decipher organization of the corresponding gene.

### 2.3. Identification of urotensin II receptors

*Xenopus laevis utr* gene predictions were identified in the Xenbase browser and the National Center for Biotechnology Information (NCBI) genome resource (https://www.ncbi.nlm.nih.gov/genome/) by using the *X. tropicalis utr* gene sequences [4] as queries.

In the present study, we used the phylogenetic nomenclature initially proposed by Tostivint *et al*. [3] and more recently taken up by Konno *et al*. [4] and Cui *et al*. [5]. According to this nomenclature, the only mammalian Utr is Utr1, while Utr4 is the counterpart of zebrafish Uts2r3 and Utr2 is the Utr subtype missing in *X. tropicalis* (table 1).

### 2.4. Phylogeny and synteny analyses

A set of 47 vertebrate UII and Urp precursor sequences was collected from NCBI and Ensembl databases and supplemented by the sequences characterized in the present study. All sequences were aligned using the Clustal algorithm [24] then manually adjusted. Molecular phylogenetic relationships were analysed using the neighbour-joining (NJ) algorithm [25] with MEGA6 software [26]. The reliability of phylogenetic trees was assessed with bootstrapping (1000 iterations). The accession numbers of the sequences used in the phylogenetic analysis are shown in the electronic supplementary material, figure S1. Ensembl database (http://www.ensembl.org/index.html) and Genomicus [27] (version 100.1, https://www.genomicus.biologie.ens.fr/genomicus-100.01/cgi-bin/search.pl) were used to determine the conserved syntenic pattern of *urp2* gene in the western clawed frog (*X. tropicalis*), chicken (*Gallus gallus*), human (*Homo sapiens*), spotted gar (*L. oculatus*) and zebrafish (*D. rerio*).

### 2.5. Gene expression analysis

#### 2.5.1. RNA extraction and cDNA synthesis from embryos and tadpoles

*Xenopus laevis* embryos and tadpoles at various developmental stages (NF1, 10, 21, 24, 37, 41 and 50, according to [28] were anaesthetized with 0.01% MS-222, rinsed in sterile water then transferred in Sorenson tubes containing 100 µl of lysis solution (provided in RNAqueous micro kit—see below). Three biological replicates were collected for each developmental stage. Tubes were flash-frozen in liquid nitrogen and stored at −80°C prior to RNA extraction. RNA was extracted using RNAqueous-Micro Total RNA Isolation Kit (Ambion, ThermoFisher) following the manufacturer's instructions. The concentration of RNA was determined using a spectrophotometer (Nanodrop TermoScientific). Next, an Agilent bio-analyser was employed to verify the quality of the collected RNA. Only samples with RNA Integrity Numer (RIN) above 7.5 were included for further processes. After, reverse transcription was conducted on 500 ng of RNA using a high capacity cDNA RT kit (Applied BioSystem) following the manufacturer's instructions (in a total 20 µl reaction). cDNA was a 20-times diluted (5 µl of cDNA in 95 µl of nuclease-free water) then stored at −20°C until use.

#### 2.5.2. RNA extraction and cDNA synthesis from tissues of juvenile frogs

Total RNA was extracted from various tissues (brain, spinal cord, eye, skin, skeletal muscles, lung, heart, liver, spleen, stomach, intestine, kidney, ovary and testis) of six juvenile frogs and purified by using RNABLE (Eurobio). Samples were treated with DNase I (Roche) to remove potential contamination by genomic DNA and then purified with phenol/chloroform extraction. The integrity of RNA was assayed by electrophoresis on a 1% agarose gel. For each tissue, 0.5 µg of total RNA was reverse transcribed using the GoScript reverse transcriptase (Promega) with random primers.

#### 2.5.3. Real-time quantitative PCR

Real-time quantitative PCR (RT-qPCR) was carried out using QuantStudio 6 flex (Life Technologies) on 384 well-plates, with a standard reaction per well containing 1/20 diluted cDNA as template (1 μl per well) plus 5 μl of mix (Power SYBR Green PCR Master Mix, Applied BioSystem). For each sample, the RT-qPCR reaction was conducted in duplicates. Water and no-template controls were used as negative controls for each primer set. The following cycling conditions were used: 1 cycle of 95°C for 2 min, followed by 45 cycles of 95°C for 15 s and 60°C for 30 s. RT-qPCR data were analysed using the QuantStudio 6 and 7 Flex RT PCR System (Life Technologies). Cycle threshold (Ct) values were obtained using auto baseline and applied to all amplicons of the same primer set. Reference genes used to normalize measurements were as follows*: SUB1 homolog (sub1-L)* and *solute carrier family 35 member B1 (slc35b1-L)* for developmental analysis and *ribosomal protein S13* (*rps13*) for tissue analysis in accordance with previous studies performed by [29] and [4], respectively. Primer sequences are listed in the electronic supplementary material, table S1. Plot was made using R software [30] (available in https://www.R-project.org/) and ggplot2 package [31].

### 2.6. *In situ* hybridization

#### 2.6.1. Riboprobe synthesis

To generate the *X. laevis urp2-*L RNA probe, the 5′RACE *urp2-*L cDNA previously cloned into pGEM-T was used as a template. To generate the four *X. laevis utr-*L receptor RNA probes, partial sequences of their cDNAs were cloned into PGEM-T after RT-PCR amplification of the corresponding transcripts then used as templates. The same procedure was followed to produce the *pkd2l1* probe (see electronic supplementary material, table S1 for primer sequences). Sense and antisense digoxigenin (Dig)-labelled probes were synthesized from the linearized plasmids with T7 or Sp6 RNA polymerases and RNA Labelling mix (Roche).

#### 2.6.2. Tissue preparation and hybridization procedures

Embryos (at stages NF29–30) and juvenile frogs were killed in 1% MS-222. Whole-mount embryos and dissected brains and spinal cords were fixed overnight at 4°C in 4% paraformaldehyde (PFA) in phosphate-buffered saline (PBS) and stored in 100% methanol at −20°C. Fixed embryos were rehydrated through a descending series of methanol solutions then bleached in 16.25% H_2_O_2_, 8.33% formamide and 0.83 × saline sodium citrate (SSC) buffer for 10 min. Fixed tissues were cryopreserved in 30% sucrose/PBS at 4°C. After, they were embedded in OCT compound (Tissue-Tek, Ted Pella, Inc.) and sectioned into 40 μm-thick slices at −20°C using a Leica CM3050 cryostat (Leica Microsystems).

In situ hybridization of whole embryos was performed as described previously [16]. Briefly, samples were prehybridized in hybridization buffer (50% formamide, 5 × SSC, 0.1 mg ml^−1^ heparin, 10 mg ml^−1^ yeast RNA, 0.1% Tween) for 2 h at 65°C. Hybridization was performed overnight at 65°C in a hybridization buffer containing the heat-denatured Dig-labelled riboprobes. The next day, samples were washed twice in 50% formamide/2 × SSC for 30 min at 37°C, twice in 2 × SSC for 15 min at 60°C and then twice in 0.2 × SSC plus 0.1% Tween 20 for 30 min at 60°C. Samples were blocked with blocking buffer (15% normal goat serum (Sigma-Aldrich), in PBS, 0.1% Tween 20) for 2 h and incubated overnight at 4°C with alkaline phosphatase-conjugated anti-Dig antibody (Roche) diluted 1 : 2500 in blocking buffer. On the third day, the enzymatic activity was revealed by the addition of BM Purple (Roche). Whole-mount embryos were post-fixed, then photographed with MZ12.5 stereomicroscope (Leica) and stored in 80% glycerol at 4°C.

In situ hybridization of floating sections was carried out with the following modifications. The hybridization buffer contained 50% formamide, 5× SSC, 50 μg ml^−1^ heparin, 0.5 mg ml^−1^ yeast RNA, 9.2 mM citric acid pH 6.0 and 0.1% Tween-20. The blocking buffer contained 2.5% blocking reagent (Sigma-Aldrich) and 5% sheep serum in maleate buffer (100 mM maleic acid, 250 mM NaCl, pH 7.5). Alkaline phosphatase-conjugated anti-Dig antibody was diluted 1 : 4400 in blocking buffer. After the revelation, sections were dehydrated, mounted in Eukitt (Sigma), then photographed with DM5500 light microscope (Leica).

### 2.7. CRISPR/Cas9-mediated genome editing

#### 2.7.1. Small guide RNA design and injection

Knock-out frogs were generated by CRISPR/Cas9 genome editing and thereafter are called as crispants (CRISPR-mediated mutants). CRISPR target sequences (20-nucleotide sequence followed by a protospacer adjacent motif (PAM) or ‘NGG’) were designed by using an online CRISPR design tool from Integrated DNA Technologies (IDT, https://eu.idtdna.com/pages) and crRNA ordered from IDT (electronic supplementary material, table S1). To prepare sgRNA, crRNA was duplexed with tracrRNA according to the manufacturer instructions.

For the *utr4* knock-out, two sgRNAs were designed between transmembrane domains 2 and 4 in order to frame the ligand-binding domain of the receptor [11]. Given the occurrence of two *utr4* homoeologues in *X. laevis* [32]*,* located on chromosomes 7L and 7S, respectively, the sgRNAs were designed so that there was not more than one mismatch between the sgRNA target site and the sgRNA sequence on either of the homoeologues (see electronic supplementary material, figure S2). The two sgRNAs were used simultaneously. To screen knock-out animals, an additional sgRNA was designed to disrupt *tyrosinase* (*tyr)*, a gene required for pigmentation. Knock-out of this gene using either CRISPR/Cas9, ZFNs or TALENs results in albinism in *Xenopus* [33–35], making the detection of knock-out animals apparent. Before injection, sgRNA (20 µM) were added to Cas9 protein (30 µM) to obtain a Cas9 : sgRNA ratio of 1 : 2 and incubated for 10 min at 37°C.

One-cell stage *Xenopus* embryos were injected with approximately 10 nanolitres of a solution containing three different Cas9-sgRNA complexes; 2 sgRNAs targeting both homoeologues of *utr4* together with one sgRNA targeting the *tyrosinase* gene. During subsequent development, abnormal and dead embryos (mainly due to gastrulation defects) were removed in order to conduct morphological phenotyping.

#### 2.7.2. Detection and sequencing of mutations in injected tadpoles

In a first test designed to check the sgRNAs, genomic DNA was extracted from tail clips of 10 tadpoles in 50 mM NaOH for 15 min at 95°C then neutralized with 1/10 volume of Tris 10 mM pH 8. All the samples were then pooled. To evaluate the efficiency of targeted deletion guided by the sgRNA pair, PCR analyses were carried out from this pooled DNA using primers flanking the targeted regions. Wild-type and truncated genomic fragments were resolved by gel electrophoresis. In order to search for mutations at the target sites of each sgRNA, non-truncated fragments were purified, sequenced on both strands then analysed by tracking indels by Inference of CRISPR Edits (ICE; Synthego, https://www.synthego.com/products/bioinformatics/crispr-analysis). After validation of the sgRNAs, the same test was performed on genomic DNA from 10 new tadpoles, but this time dealt separately, to determine the relationship between their genotype and phenotype.

### 2.8. Phenotype analysis of *utr4* crispants

#### 2.8.1. Body curvature analysis

**Figure 10.**
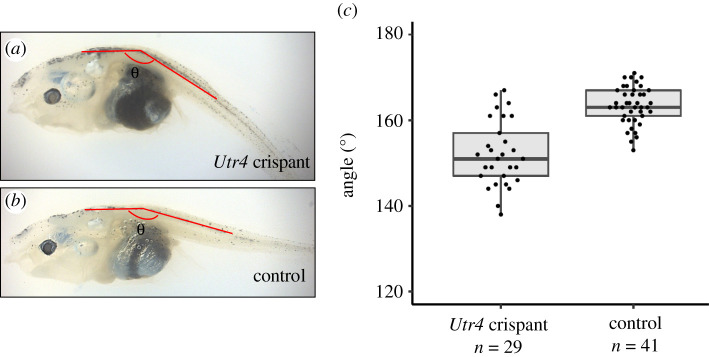
The phenotypic effect of *utr4* knock-out. Still images showing the curved body phenotype of *utr4* crispant (*a*) and control (*b*) tadpoles. (*c*) Statistical analysis of the angle of body curvature of *utr4* crispant tadpoles compared to control tadpoles at stages NF46 to NF52. *n* = 41 controls and 29 *utr4* crispants, ****p < 0.0001.

*Xenopus laevis utr4* crispant and control tadpoles (from stages NF46 to 52) were euthanized, fixed in 4% PFA, stored in 100% methanol and then re-hydrated in PBS. Tadpoles were imaged in a bright field with a Lumenera Infinity *3*-*6*UR microscope camera attached to an Olympus Microscope operated with Infinity Analyze software in an agarose plate submerged in PBS laterally with the head pointing to the left. To define the body curvature, the images were analysed with the Fiji Angle tool. The line was drawn starting from the posterior half of the tail mid line to the head (figure 10), and the value of the angle formed in between the two lines was defined by the Angle tool for each tadpole.

To evaluate the statistical difference in body curvature in *utr4* crispant and control tadpoles, the measured angles were applied to Student's *t*-test (function t.test, two-tailed, unequal variance) using Microsoft Excel 2020 (function t.test, two-tailed, unequal variance). Tadpoles were considered as curled down when the head-to-tail angle was less than 160°.

#### 2.8.2. Quantification of locomotor activity

To record the locomotion of tadpoles, we used a Phantom Miro M110 camera (Vision Research, Elancourt, France) set at 700 or 1000 frames per second (s). Stage NF53 tadpoles were placed in a large Petri dish (30 cm diameter with 3 cm of water depth), stimulated to swim and recorded for 3 s. We recorded five *utr4* crispant and three control tadpoles. Three videos per individual were recorded, saved and analysed using ProAnalyst (Xcitex, Cambridge, MA, USA) software. For each frame, the eye was digitized either manually or using the auto-tracking routine implemented in ProAnalyst. Raw coordinates were exported to Excel and smoothed using a zero phase-shift low-pass Butterworth filter with a cut-off frequency set at 50 Hz. Next, we calculated the cumulative distance swum along the path. Instantaneous velocities and accelerations were calculated by numerical differentiation of the smoothed cumulative displacement profile. We extracted peak velocity, acceleration and deceleration from the kinematic data and further calculated the straight-line distance between the beginning and the end of the video recording. We calculated the sinuosity of the trajectory by dividing the cumulative distance by the straight-line distance. All variables were log_10_-transformed and analysed using IBMSPSS statistics (v. 26). We used univariate analyses of variance to test for differences in swimming kinematics between *utr4* crispant and control tadpoles for each variable tested.

## 3. Results

### 3.1. Structure of Urp2 precursor cDNAs and genes in *Xenopus tropicalis* and *Xenopus laevis*

**Figure 1.**
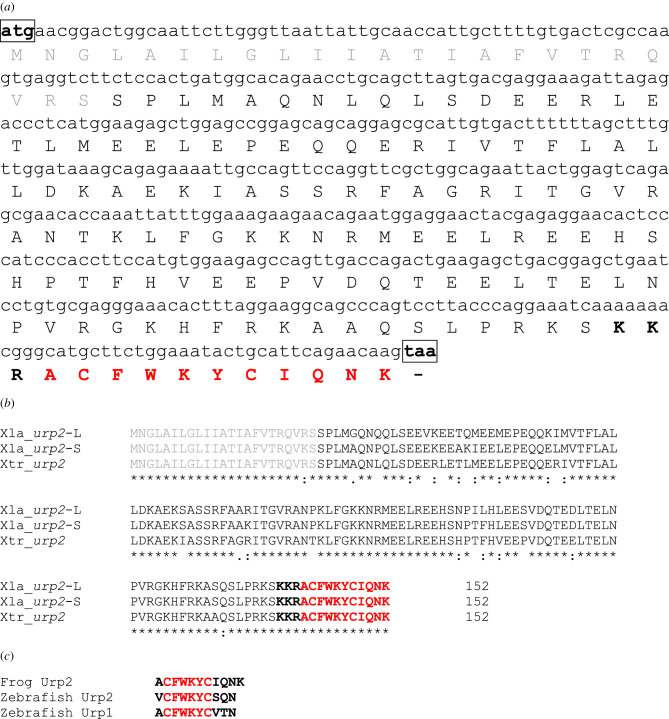
Primary structure of frog Urp2 precursors. (*a*) Nucleotide sequence of the *X. tropicalis urp2* cDNA and deduced amino acid sequence. (*b*) Alignment of the aa sequences of *X. tropicalis* and *X. laevis* prepro-Urp2. An asterisk denotes conserved residues. Precursor sizes are indicated. Xtr, *X. tropicalis*; Xla, *X. laevi*s. (*c*) Comparison of Urp2 and Urp1 primary sequences in *Xenopus* and zebrafish. Initiation (ATG) and stop codons are boxed. Signal peptides are in grey. Putative bioactive peptides are highlighted in red. Cleavage sites are in bold. The sequences of the two *X. laevis* Urp2 cDNAs have been deposited in the GenBank database under the accession number MZ054702 and MZ054703, respectively.

The nucleotide and deduced amino acid sequences of the *X. tropicalis urp2* cDNA are shown in figure 1*a*. The coding region of this cDNA consists of 456 base pairs (bp) that encode a 152 amino acid (aa) protein. The primary structure of the protein, called prepro-Urp2, encompasses a 23-aa putative signal peptide [36], a 115-aa cryptic sequence, a 3-aa proteolytic cleavage site KKR and the Urp2 peptide sequence ACFWKYCIQNK. Comparison of the cDNA with genomic sequence revealed that the *X. tropicalis urp2* gene is composed of 5 exons and 4 introns (electronic supplementary material, figure S3). The fifth exon fully encodes the putative mature peptide Urp2, as in zebrafish [16].

In *X. laevis*, two distinct *urp*2 cDNAs were found (electronic supplementary material, figure S4). Both cDNAs were called *urp2*-L and *urp2*-S on the basis of the chromosome location of the corresponding genes (chromosome 2L and 2S, respectively) [32]. *X. laevis* prepro-Urp2-L and Urp2-S exhibit a high level of sequence identity with *X. tropicalis* prepro-Urp2 (approx. 86–91% sequence identity for each; figure 1*b*; electronic supplementary material, table S2). The primary sequence of Urp2 is the same for both precursors. As *X. tropicalis urp2* gene, *X. laevis urp2*-L and -S genes are composed of 5 exons and 4 introns (electronic supplementary material, figures S5 and S6).

### 3.2. Comparison of prepro-Urp2 sequences from other species

Frog and fish Urp2 precursors display only low-sequence identity (from 21.7 to 29.0%, depending on the species, electronic supplementary material, table S2). They do not exhibit appreciable sequence similarities outside the Urp2 domain (electronic supplementary material, figure S7). Frog Urp2 contains one more aa residue than fish Urp2 and it differs at two additional positions (1 and 8) (figure 1*c*). Frog Urp2 differs from fish Urp1, which is one residue shorter [2], at positions 8 and 9 (figure 1*c*).

### 3.3. Identification of *Xenopus laevis* urotensin II receptors

As in *X. tropicalis* [4], four Utr subtypes were identified in *X. laevis*, namely Utr1, Utr3, Utr4 and Utr5, each of them consisting of two homoeologues (L and S) (electronic supplementary material, figures S8 and S9). It is noteworthy that Utr3-S and Utr4-S (but not Utr3-L and Utr4-L) lack multiple transmembrane domains (electronic supplementary material, figure S9).

### 3.4. Phylogenetic analysis of the UII and Urp precursors

**Figure 2.**
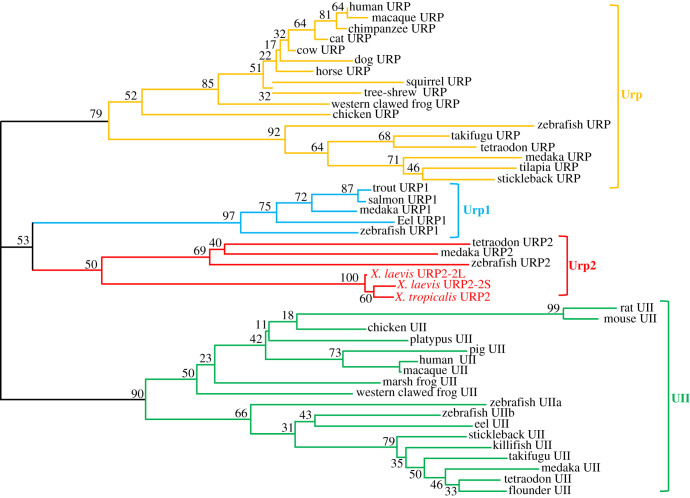
Phylogenetic tree of vertebrate UII-Urp precursor sequences. Phylogenetic analysis of 47 vertebrate prepro UII-Urp amino acid sequences was performed using the NJ distance-based method, with 1000 bootstrap replicates. The number shown at each branch node indicates in percentage the bootstrap value. Sequence references and alignment are given in the electronic supplementary material, figures S1 and S10, respectively.

Based on an amino acid alignment of 47 selected UII and Urp precursor sequences (electronic supplementary material, figure S10), a phylogenetic tree was constructed using the NJ distance-based method [25]. As depicted in figure 2, the phylogenetic tree segregated the UII and Urp sequences into four main clades which correspond to the four paralogues UII, Urp, Urp1 and Urp2. Bootstrap support values for these groups were 90, 79, 97 and 50%, respectively.

### 3.5. Synteny analysis of the *urp2* genes in vertebrates

**Figure 3.**
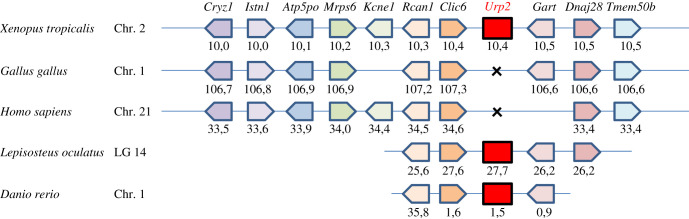
Synteny of genes in the *urp2* locus in five selected osteichthyan species: human (*H. sapiens*), chicken (*G. gallus*), western clawed frog (*X. tropicalis*), spotted gar (*L. oculatus*) and zebrafish (*D. rerio*). Genes are represented by block arrows. The position of the genes (in megabases, Mb) is displayed below each box, according to the Ensembl database. The detailed chromosomal locations of genes displayed in this map are included in the electronic supplementary material, table S3.

To further resolve the orthologue relationship between fish and frog *urp2* genes, a synteny analysis was performed. For this purpose, the genomic environment of *urp2* genes was determined in *X. tropicalis* and compared to that of various representative osteichthyan species, namely human, chicken, spotted gar and zebrafish. As shown in figure 3, the three closest genes to *urp2* in *X. tropicalis*, *rcan1*, *clic6* and *gart* are also located in the vicinity of *urp2* in ray-finned fishes (see also electronic supplementary material, table S3 for more details).

### 3.6. *Urp2* gene expression

#### 3.6.1. Urp2 gene expression during *Xenopus* laevis development

**Figure 4.**
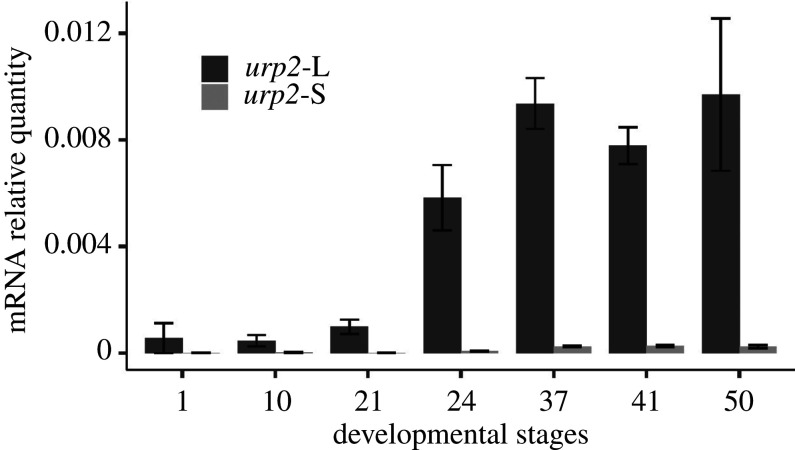
*urp2*-L and *urp2*-S gene expression during *X. laevis* development. Relative mRNA expression levels were measured by quantitative RT-PCR and represented as ratios to the mRNA levels of the housekeeping genes *sub1-L* and *slc35b1-L*.

Gene expression profiles of *urp2-*L and *urp2*-S genes were examined by RT-qPCR during *X. laevis* development from fertilization (NF1) to pre-metamorphosis (NF50). mRNA level of *urp2*-L was found to strongly increase from NF21 to NF37 and became roughly stable thereafter (figure 4). Almost no *urp2*-S mRNA was detected at the different stages examined.

#### 3.6.2. Tissue expression of *urp2* in *Xenopus laevis* frog

**Figure 5.**
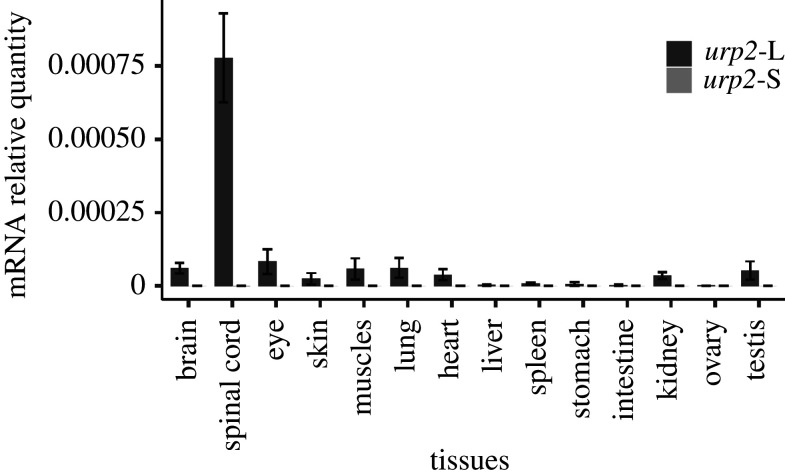
The tissue expression of *urp2*-L and *urp2*-L genes in juvenile *X. laevis* frog*.* Relative mRNA expression levels were measured by quantitative RT-PCR and represented as ratios to the mRNA level of the housekeeping gene *rps13*. Values are estimated for samples from six frogs.

RT-qPCR was used to determine the distribution of *urp2-*L and *urp2*-S mRNAs in different tissues of juvenile frogs (figure 5). The highest amount of *urp2-*L mRNA was measured in the spinal cord. *urp2-*L mRNA was also detected in the brain, eye, skin, muscles, lung, heart, kidney and testis but at a much lower level. In all other tissues, *urp2-*L expression was very low or undetectable. Almost no *urp2*-S mRNA could be detected in the tissues examined.

#### 3.6.3. Localization of *urp2* mRNA in *Xenopus laevis* embryos

**Figure 6.**
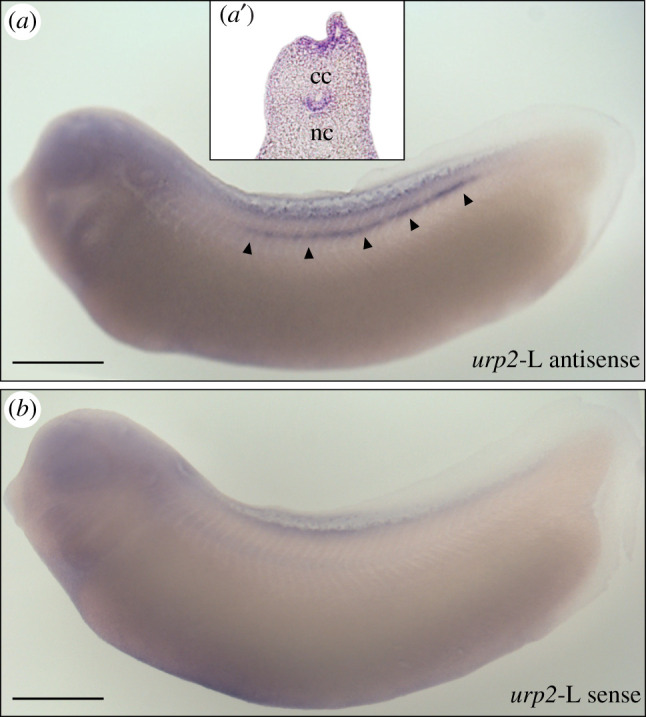
Localization of *urp2*-L mRNA in *X. laevis* embryo revealed by *in situ* hybridization. Lateral view of embryos (stage NF29-30) hybridized with antisense (*a*) and sense (*b) urp2*-L probes. Staining of cells contacting the central canal is indicated by arrowheads. (*a’*), detail of (*a*) in the transverse section. cc, central canal; nc, notochord. Scale bars: 450 µm.

The localization of *urp2*-L mRNA was studied in *X. laevis* embryos (NF29-30) by *in situ* hybridization. As depicted in figure 6*a*, the *urp2*-L staining was mainly detected along the ventral part of the spinal cord. At higher magnification, *urp2*-L*-*positive cells were observed ventrally to the central canal (figure 6*a*′). No staining was detected in the spinal cord with the sense *urp2*-L riboprobe (figure 6*b*).

#### 3.6.4. Localization of *urp2* mRNA in the central nervous system of *Xenopus laevis* frog

**Figure 7.**
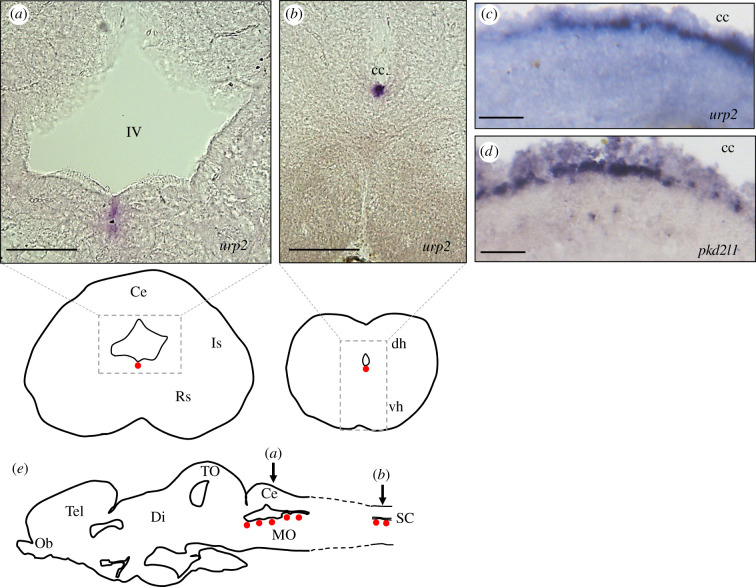
Localization of *urp2*-L mRNA in the brain and spinal cord of juvenile *X. laevis* frog revealed by *in situ* hybridization. Transverse sections (40 µm) of juvenile frog hindbrain (*a*) and spinal cord (*b*) hybridized with antisense *urp2*-L probe. (*c*,*d*) Sagittal sections (40 µm) of juvenile frog spinal cord hybridized with antisense *urp2*-L (*c*) and *pkd2l1* (*d*) probes. cc, central canal; IV, fourth ventricle. (*e*) Schematic sagittal section depicting the distribution of *urp2* mRNA hybridization signals (*red dots*). The level of the coronal sections in (*a*) and (*b*) is indicated by arrows. Ce, cerebellum; dh, dorsal horn of the spinal cord; Di, diencephalon; Is, isthmic nucleus; MO, medulla oblongata; Ob, olfactive bulbs; Rs, nucleus reticularis superior; SC, spinal cord; Tel, telencephalon; TO, tectum opticum; vh, ventral horn of the spinal cord. Anatomical structures are designated according to [37]. Scale bars: 150 µm (*a*,*b*), 50 µm (*c*,*d*).

The expression of the *urp2* gene in the brain and spinal cord was studied in juvenile frogs by *in situ* hybridization. In the brain, the *urp2*-L staining was observed in the ventral midline of the medulla oblongata, ventrally to the fourth ventricle (figure 7*a*). In the spinal cord, *urp2*-L*-*expressing cells were seen ventrally to the central canal (figure 7*b*). As depicted in figure 7*c*,*d*, spinal *urp2*-L mRNA-containing cells occupy the same position as cells expressing *pkd2l1*, a specific marker for CSF-contacting neurons [38].

### 3.7. *Utr* gene expression

#### 3.7.1. *Utr* gene expression during *Xenopus laevis* development

**Figure 8.**
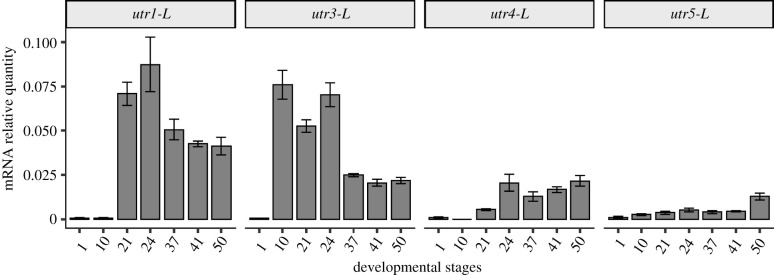
*utr* gene expression during *X. laevis* development. Relative mRNA expression levels were measured by quantitative RT-PCR and represented as ratios to the mRNA levels of the housekeeping genes *sub1-L* and *slc35b1-L*.

Gene expression profile of *utr* genes (*utr1*-L*, utr3*-L*, utr4*-L and *utr5*-L) was examined by RT-qPCR during *X. laevis* development from NF1 to NF50 (figure 8). *Utr1*-L mRNA level strongly increased from NF10 to NF21 and appeared a bit lower between NF37 and NF50. *Utr3*-L displayed a similar expression profile to that of *utr1*-L, but with a slightly earlier phase of increase. *utr4*-L mRNA level increased from NF10 to NF 24 and became roughly stable thereafter. *utr5*-L mRNA level stayed very low until NF41 then slightly increased between NF41 and NF51. *utr*-S mRNA levels were very low at all stages examined (data not shown).

#### 3.7.2. Localization of urotensin II receptors mRNAs in *Xenopus laevis* embryos

**Figure 9.**
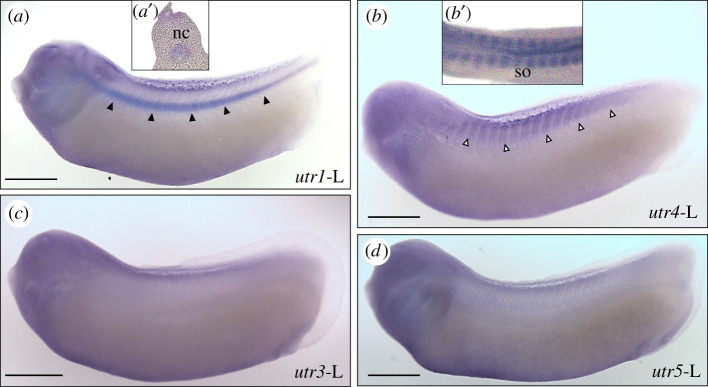
Localization of urotensin II receptor (*utr*) mRNAs in *X. laevis* embryo revealed by *in situ* hybridization. Lateral view of embryos (stages NF29-30) hybridized with antisense *utr1*-L (*a*), *utr4*-L (*b*), *utr3*-L (*c*) and *utr5*-L (*d*) antisense RNA probes. (*a′*) and (*b′*) are details of (*a*) and (*b*), in dorsal view and transverse sections, showing the staining in notochord (nc, filled arrowheads) and somites (so, empty arrowheads), respectively. Scale bars: 450 µm.

The localization of urotensin II receptor mRNAs was studied in *X. laevis* embryos (NF29-30) by *in situ* hybridization (figure 9). The *utr1*-L staining was mainly detected in the notochord (figure 9*a,a*′), while the *utr4*-L staining was primarily present in dorsal somites (figure 9*b,b*′). It is noteworthy that in juvenile frogs, *utr4*-L expression was very low in skeletal muscles (electronic supplementary material, figure S11). No apparent staining was observed with *utr2*-L (figure 9*c*) and *utr5*-L (figure 9*d*) antisense riboprobes (table 1).

### 3.8. *Utr4* crispants

#### 3.8.1. Generation and phenotype analysis

In zebrafish, mutants for *uts2r3* (*utr4* counterpart) lead to spine deformation in larvae and adults [12]. To test whether the function of the Utr4 signalling pathway is conserved in *X. laevis*, we produced CRISPR/Cas9-mediated mutant (i.e. crispants). We designed two RNA guides, each targeting both *utr4*-L and *utr4*-S, and injected them into fertilized eggs together with a guide RNA against the *tyrosinase (tyr)* gene as a control for injection efficiency (see Material and methods; electronic supplementary material, figure S2). The efficiency of *utr4* disruption was assayed by PCR using primers flanking the targeted region. As shown in the electronic supplementary material, figure S12, expected deleted fragments were detected from both *utr4*-L and *utr4*-S at 320 and 560 bp, respectively. Moreover, the analysis of Sanger trace data from non-deleted PCR products showed that the knock-out score (percentage of frameshifting indels) at the two targeted sites were 39% and 71% for *utr4*-L and 61% for both sites for *utr4*-S (see electronic supplementary material, table S4). Thus, on average, 82% of non-deleted *utr4*-L copies were disrupted (85% for *utr4*-S). Altogether these results show an efficient disruption of *utr4*.

Embryos knocked-out for both *utr4* and *tyr* (*utr4* crispants) developed normally until stage NF40-42. Starting NF42, an abnormal curvature of the antero-posterior axis was observed (figure 10*a,b*). While in control tadpoles (invalidated for only *tyr*), the average angle between the head and the body was 164° (±4.3° standard deviation, s.d., *n* = 41), this angle was only 153° (s.d. 7.7, *p* < 0.001, *n* = 29) in *utr4* crispants (figure 10*c*).

Electronic supplementary material, figure S13 depicts the relationship between genotype and phenotype of 10 tadpoles randomly selected among the 29 *utr4* crispants mentioned above. The results show that (*i)* unmutated or slightly mutated tadpoles exhibit a wild-type phenotype and (*ii)* all tadpoles that exhibit a curved phenotype are mutated with either medium or strong mutation level. Taken together, these results strongly suggest that the curved phenotype is caused by *utr4* disruption. The occurrence of some mutated crispants without abnormal curvature could be due to mosaicism.

#### 3.8.2. Quantification of locomotor activity

**Figure 11.**
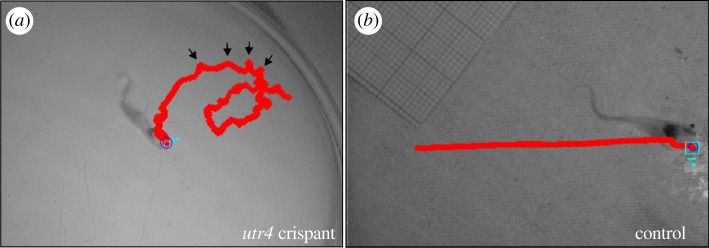
Figure illustrating the tracking of a sequence of an *utr4* crispant (*a*) and a control (*b*) tadpole. Noticeable is the erratic trajectory of the *utr4* crispant (in red) characterized by long-axis rotations visible as marked deviations from the straight-line path (illustrated by the arrows for part of the trajectory) in contrast with the rectilinear displacement of the control tadpole.

A summary of the kinematic variables is provided in table 2. Swimming in *utr4* crispant tadpoles was erratic and showed regular long-axis rotations (figure 11). This resulted in a greater cumulative displacement along the path in crispants (*F*_1,18_ = 10.22; *p* = 0.005; table 2) while swimming a similar straight-line distance (*F*_1,18_ = 1.19; *p* = 0.29). Although crispants had a greater sinuosity (table 2), the difference was not statistically significant (*F*_1,18_ = 2.62; *p* = 0.12). Swimming in crispants was further characterized by higher mean (*F*_1,18_ = 4.60; *p* = 0.046) and peak velocity (*F*_1,18_ = 8.42; *p* = 0.01) and higher peak accelerations (*F*_1,18_ = 4.45; *p* = 0.049) and decelerations (*F*_1,18_ = 4.91; *p* = 0.04).

**Table 2.** Mean kinematic variables of *utr4* crispants. Table entries are means ± s.d. Significant differences (*p* < 0.05) are in italics.

	wild type	knock-out
straight-line distance (mm)	55.04 ± 46.46	70.53 ± 39.83
*cumulative distance* (*mm*)	*69.80* **±** *54.18*	*231.15* **±** *204.94*
sinuosity	1.43 ± 0.42	6.02 ± 7.97
*peak velocity* (*mms^−1^*)	*120.56* **±** *138.65*	*3856.03* **±** *9785.67*
*mean velocity* (*mms^−1^*)	*22.10* **±** *17.74*	*61.58* **±** *58.32*
*peak acceleration* (*ms^−2^*)	*14.14* **±** *16.95*	*568.07* **±** *1451.47*
*peak deceleration* (*ms^−2^*)	*−12.86* **±** *17.95*	**−***569.74* **±** *1460.51*

## 4. Discussion

The present study reports the characterization of *urp2* genes in the *Xenopus* genus. *X. tropicalis* possesses a single *urp2* gene, while two homoeologues are present in *X. laevis*, in agreement with the pseudotetraploid status of this species [32]. So far, *urp2*, first identified in the Japanese eel [15] and zebrafish [16] was only known in ray-finned fishes [2]. Therefore, as far as we are aware of, our study provides the first conclusive evidence for the presence of *urp2* in tetrapods.

The molecular organization of the Urp2 precursor is the same in all species examined, with a signal peptide followed by a long central segment, a conserved pair of basic residues and, finally, the Urp2 sequence at their C-terminus. In fish, the Urp2 sequence encompasses ten aa with a fully conserved cyclic region at position 2–7 followed by three residues SQN [15,16]. In frogs, the Urp2 contains one more residue at its C-terminus which exhibits the original IQNK extension. Despite these differences, the orthology between fish and frog *urp2* genes is clearly supported by phylogenetic and synteny analyses.

It has been previously shown that *urp2* arose through the two whole-genome duplication events (1R and 2R) that took place early during vertebrate evolution [2,16]. The occurrence of *urp2* in both fish and amphibians is in full agreement with this model. Moreover, its absence in both chicken and mammals suggests that it has been subsequently lost in the amniote lineage. It is noteworthy that the *urp1* gene, which is thought to have appeared at the same time as *urp2* in 2R [2,16] could not be found in the frog's genome.

In zebrafish, *urp2* is primarily expressed in the hindbrain and spinal cord, both in the embryo and the adult. In the spinal cord, *urp2* mRNA was located in a small population of sensory neurons called CSF-contacting neurons while in the brainstem, *urp2*-expressing cells were found at the ventral edge of the fourth ventricle [16,17]. The present study reveals that this expression pattern is well conserved in *X. laevis*, since *urp2* mRNA-containing cells also expressed *pkd2l1*, a specific marker for CSF-contacting neurons [38]. *urp2* expression was apparent in spinal CSF-contacting neurons well before hatching and remained visible in the same cell type beyond metamorphosis when tadpoles turn into frogs. It should be noted that only the *urp2*-L transcript could be significantly detected in *X. laevis* tissues suggesting that the *urp2-*S copy is very little expressed and therefore may become pseudogenized either in the near or more distant future [39].

Urp1 and Urp2 have been recently shown to be required for correct axis formation and maintenance in zebrafish. The knock-down of *urp1* leads to a curled down axis in zebrafish embryos, while its overexpression leads to the opposite curvature [12]. Although the knock-down of *urp2* has no effect in wild-type embryos, its overexpression, as that of *urp1,* can rescue body axis defects in ciliary mutants, suggesting that Urp1 and Urp2 actions are largely redundant [12]. These actions are thought to be mediated by Uts2r3, a member of the Utr family specifically expressed in muscles, since the *uts2r3* knock-down also produced body curvature in zebrafish embryos [12]. Based on of these data, it has been hypothesized that *uts2r3* mediated-contractile activity of dorsal muscle brings about proper axial morphogenesis [12].

Recently, Konno *et al*. [4] reported the occurrence of four Utr subtypes in *X. tropicalis* (Utr1, Utr3, Utr4 and Utr5), instead of five in zebrafish [5]. In agreement with these findings, we showed that four Utr homoeologue pairs are also present in *X. laevis*. Utr3-S and Utr4-S were found to lack multiple transmembrane domains, suggesting that they are not functional. Generally, *utr-S* genes are all little expressed suggesting that, as *urp2*-S, they may become pseudogenized.

We identified *X. laevis* Utr4-L as the frog counterpart of zebrafish Uts2r3, evolutionarily and functionally. Thus, as in zebrafish, *utr4-L* is specifically expressed in the dorsal somitic musculature of the *X. laevis* embryo. Moreover, the gene knock-out of *utr4* resulted in a severe axial curvature of the tadpoles. The first signs of the spinal axis deformation in *utr4* crispants could not be detected until stage NF42, which is consistent with the fact that *utr4* expression peaks shortly before hatching. Overall, the phenotype of *Xenopus* *utr4* crispants is reminiscent to that of zebrafish *uts2r3* mutants.

The curvature of the body axis in the vertical plane in *utr4* crispant tadpoles significantly impacted their locomotion. Swimming in crispants was erratic and included significant long-axis rotation leading to a somewhat higher sinuosity and a significantly higher cumulative displacement along the path. This resulted in higher swimming speeds [40], accelerations and decelerations. However, the ultimate straight-line distance swum by the crispant tadpoles in the same time frame was not different. Consequently, *utr4* crispant tadpoles shall compensate for their off-trajectory movements by swimming faster. The increased curvature in the sagittal plane in *utr4* crispant tadpoles combined with the oscillatory movements of the tail resulted in a torque being generated causing the long-axis rotation of the tadpole and the greater cumulative distance swam by the tadpole to achieve the same straight-line displacement.

It is noteworthy that the developmental expression dynamics of *utr4* is very similar to that of *urp2*, which agrees with the idea that Urp2 is a natural ligand of Utr4 in the somatic musculature. Taken together, our results show that the role of the Utr4 signalling pathway in the control of body straightness, first reported in zebrafish, is conserved in *X. laevis*. Hence, it is likely that this pathway was already operant in the osteichthyan ancestor, more than 400 million years ago [41].

In zebrafish, the view that Utr4 can be activated by other ligands than Urp2 cannot be ruled out, while in frog, it is well supported by a recent study [4]. With this respect, the occurrence of UII and Urp in motoneurons of the *X. laevis* spinal cord [23,42] makes both peptides additional plausible candidates due to the close functional relationships between muscles and motoneurons. In this regard, it is noteworthy that in zebrafish, Urp (but not UII) is also expressed in spinal motoneurons [17,43,44]. Further studies would be needed to test the roles of Urp and/or UII in both *Xenopus* and zebrafish in the control of body straightness.

In zebrafish, the Reissner fibre has also been found to play an essential role for axis morphogenesis in the zebrafish embryo [45–47]. The Reissner fibre is an acellular thread formed by the aggregation of the SCO-spondin glycoprotein which extends caudally through the central canal of the spinal cord [48]. It has been found that *scospondin* mutant embryos lacking the Reissner fibre fail to extend a straight body axis during embryonic development [45,46]. Interestingly, the Reissner fibre appears to be required for the expression of *urp2* in CSF-contacting neurons [13,14], which is in agreement with recent findings showing that it is functionally coupled to the mechanosensory function of CSF-contacting neurons [49]. The Reissner fibre is highly conserved and present in the central canal of almost all vertebrate species [48] including *X. laevis* [50]. The role of the Utr4 pathway is therefore probably part of a strongly conserved mechanism of control of the body axis morphogenesis in vertebrates which links the Reissner fibre, the CSF-contacting neurons and the somitic musculature.

It is likely that the Utr4 signalling pathway has been lost several times in the amniote lineage since Utr4 lacks both in the chicken and mammals but still present in the lizard *Anolis carolinensis* [3,5]. Whether other molecular players now ensure the functions previously exerted by Utr4 (plus Urp1 and/or Urp2) is an important issue in mammals. Some of these players could also belong to the urotensin II-ergic system, such as Utr1, the only mammalian Utr subtype, which is strongly expressed in muscles and UII and Urp, which are mainly expressed in motoneurons [51]. In this regard, the recent findings that mutations in Utr1 (generally called Utsr2r in mammals) are associated with adolescent IS as well as the fact that *utr1* expression is significantly increased in scoliotic patients are of great importance [52].

## 5. Conclusion

In the present study, we characterized for the first time the *urp2* gene in a tetrapod. We showed that *X. laevis urp2* is mainly expressed in CSF-contacting neurons of the spinal cord and hindbrain. We also showed that its putative receptor *utr4* is primarily expressed in dorsal somites. Finally, we revealed that the *utr4* knock-out results in a severe axial curvature of the tadpoles. Taken together, our results strongly suggest that the role of the Utr4 signalling pathway in the control of body straightness is an ancestral feature of bony vertebrates and not just a peculiarity of ray-finned fishes. Understanding why this pathway has been lost in amniotes and determining how it has been replaced is an essential challenge for the future. In particular, answers to these questions should have critical applications in human pathology, notably to identify novel pathogenic mechanisms of IS [53].

## Data accessibility

Novel nucleotide sequences reported in this paper have been deposited in the GenBank database under accession nos. MZ054702 and MZ054703.
